# Comparative Profiling of Volatile Compounds in Popular South Indian Traditional and Modern Rice Varieties by Gas Chromatography–Mass Spectrometry Analysis

**DOI:** 10.3389/fnut.2020.599119

**Published:** 2020-12-09

**Authors:** Kaliyaperumal Ashokkumar, Mahalingam Govindaraj, Sampathrajan Vellaikumar, V. G. Shobhana, Adhimoolam Karthikeyan, Manoharan Akilan, Jeyaraman Sathishkumar

**Affiliations:** ^1^Crop Improvement, Cardamom Research Station, Kerala Agricultural University, Pampadumpara, India; ^2^School of Agriculture, PRIST Deemed University, Thanjavur, India; ^3^Crop Improvement Program, International Crops Research Institute for the Semi-Arid Tropics, Hyderabad, India; ^4^Agricultural College and Research Institute, Tamil Nadu Agricultural University, Madurai, India; ^5^Subtropical Horticulture Research Institute, Jeju National University, Jeju, South Korea; ^6^Department of Plant Breeding and Genetics, Agricultural College and Research Institute, Tamil Nadu Agricultural University, Madurai, India

**Keywords:** *Oryza sativa* L., traditional aromatic rice, volatile organic compounds, GC-MS analysis, total phenol

## Abstract

Rice (*Oryza sativa* L.) is one of the major cereal crops cultivated across the world, particularly in Southeast Asia with 95% of global production. The present study was aimed to evaluate the total phenolic content (TPC) and to profile all the volatile organic compounds (VOCs) of eight popular traditional and two modern rice varieties cultivated in South India. Thirty-one VOCs were estimated by gas chromatography–mass spectrometry (GC-MS). The identified volatile compounds in the 10 rice varieties belong to the chemical classes of fatty acids, terpenes, alkanes, alkenes, alcohols, phenols, esters, amides, and others. Interestingly, most of the identified predominant components were not identical, which indicate the latent variation among the rice varieties. Significant variations exist for fatty acids (46.9–76.2%), total terpenes (12.6–30.7%), total phenols (0.9–10.0%), total aliphatic alcohols (0.8–5.9%), total alkanes (0.5–5.1%), and total alkenes (1.0–4.9%) among the rice varieties. Of all the fatty acid compounds, palmitic acid, elaidic acid, linoleic acid, and oleic acid predominantly varied in the range of 11.1–33.7, 6.1–31.1, 6.0–28.0, and 0.7–15.1%, respectively. The modern varieties recorded the highest palmitic acid contents (28.7–33.7%) than the traditional varieties (11.1–20.6%). However, all the traditional varieties had higher linoleic acid (10.0–28.0%) than the modern varieties (6.0–8.5%). Traditional varieties had key phenolic compounds, stearic acid, butyric acid, and glycidyl oleate, which are absent in the modern varieties. The traditional varieties Seeraga samba and Kichilli samba had the highest azulene and oleic acid, respectively. All these indicate the higher variability for nutrients and aroma in traditional varieties. These varieties can be used as potential parents to improve the largely cultivated high-yielding varieties for the evolving nutritional market. The hierarchical cluster analysis showed three different clusters implying the distinctness of the traditional and modern varieties. This study provided a comprehensive volatile profile of traditional and modern rice as a staple food for energy as well as for aroma with nutrition.

## Introduction

Rice is a leading staple crop after wheat, and two-thirds of the world population consumed rice as their primary food source (1). The global production of rice is 769.4 m tons from 167.2 m ha (2), and it plays a critical role in food systems. Rice production in developing countries achieved a 117% yield increase through the efforts of the Green Revolution, which helped to prevent starvation in developing countries (3). Future demand for rice supply is predicted to reach 9.7 billion by 2050 for promising food and nutritional security to the growing population (4). In Asia, more than two billion people attain their 80% of energy requirement from rice grains, which covers 80% carbohydrates, 7–8% protein, 3% fiber, and 3% fat (5). Furthermore, rice is also featured in a wide range of cultural, social, and religious activities for the Asian population (6). However, modern rice varieties are often lacking in essential micronutrients compared with traditional varieties and landraces (7). Additionally, a recent study exposed that traditional rice grains accumulated a 2-fold higher folate concentration than the grains of modern varieties of rice (7).

In India, traditional rice varieties with coarse grains are generally consumed in rural areas. The production cost of traditional rice varieties is relatively lesser than modern rice varieties with fine quality grains (8). Worldwide, most people demand more of modern rice grains, which has reduced the production of traditional rice varieties; hence, increasing the consumption of traditional rice varieties might pave the way in getting more varieties of traditional rice into the market. Also, the consumption of indigenous rice varieties helps to improve the farmer's economic background.

The traditional rice varieties like Kichili samba, Seeraga samba, Kaiviral samba, Mappilai samba, Karuppu kavuni, Kattuyanam, Kuzhiyadichan, and several other varieties were grown from ancient times in southern Indian states, particularly in Tamil Nadu. The Palakkadan matta traditional rice variety is mainly grown in Palakkad district of Kerala, has bold grains with a red pericarp, and has a unique taste attributed to its geographic location (9). Traditional rice varieties like Mappilai samba, Sigappu kavuni, Karuppu kavuni, and Kattuyanam have been used for the formulation of several South Indian recipes. Furthermore, the Indian traditional rice has still been used to treat several diseases including fever, blood pressure, paralysis, leucorrhea, rheumatism, gastrointestinal problems, and skin diseases and also used for increasing the milk level in lactating women (6, 10). Traditional rice grains are also linked to anti-inflammatory and diuretic activity (11).

The rice grains are a storehouse of several predominant chemical compounds, such as phenols, sterols, flavonoids, terpenoids, anthocyanins, tocopherols, tocotrienols, oryzanol, and phytic acid (12, 13). The concentration of total phenolic content (TPC) in rice grains is positively associated with antioxidant activity (14–16). TPC is also used to control blood lipids, which help in the prevention of cardiovascular diseases (17) and diabetes (18).

Rice volatile compounds are classified into several classes, including aldehydes, alcohols, terpenes, benzenoids, amino acid and fatty acid derivatives, and others (19). The major volatile constituents contributing to the aroma of traditional varieties have been identified, viz. hexanal, octanal, non-anal, (E)-2-octenal, 1-octen-3-ol, guaicol, and vanillin (20, 21). Some of the studies were conducted to evaluate the variability of volatile constituents from dehusked traditional rice grains; however, the origin of most of the details of the samples is not sufficiently defined. Moreover, in most of the cases, they are commercial samples obtained from markets with unknown original habitat or may be mixtures of various rice varieties, which could not represent the individual chemotypes. Furthermore, the volatile profiles of rice might be a potential indicator of the uniqueness of a particular variety and used to interpret grain quality (20). In this context, the objective of the present investigation was to chemically characterize the volatile constituents of eight popular traditional rice varieties and two modern rice varieties of rice, which will aid the selection of suitable accessions based on the demands of consumers and manufacturers.

## Materials and Methods

**Figure 1:**
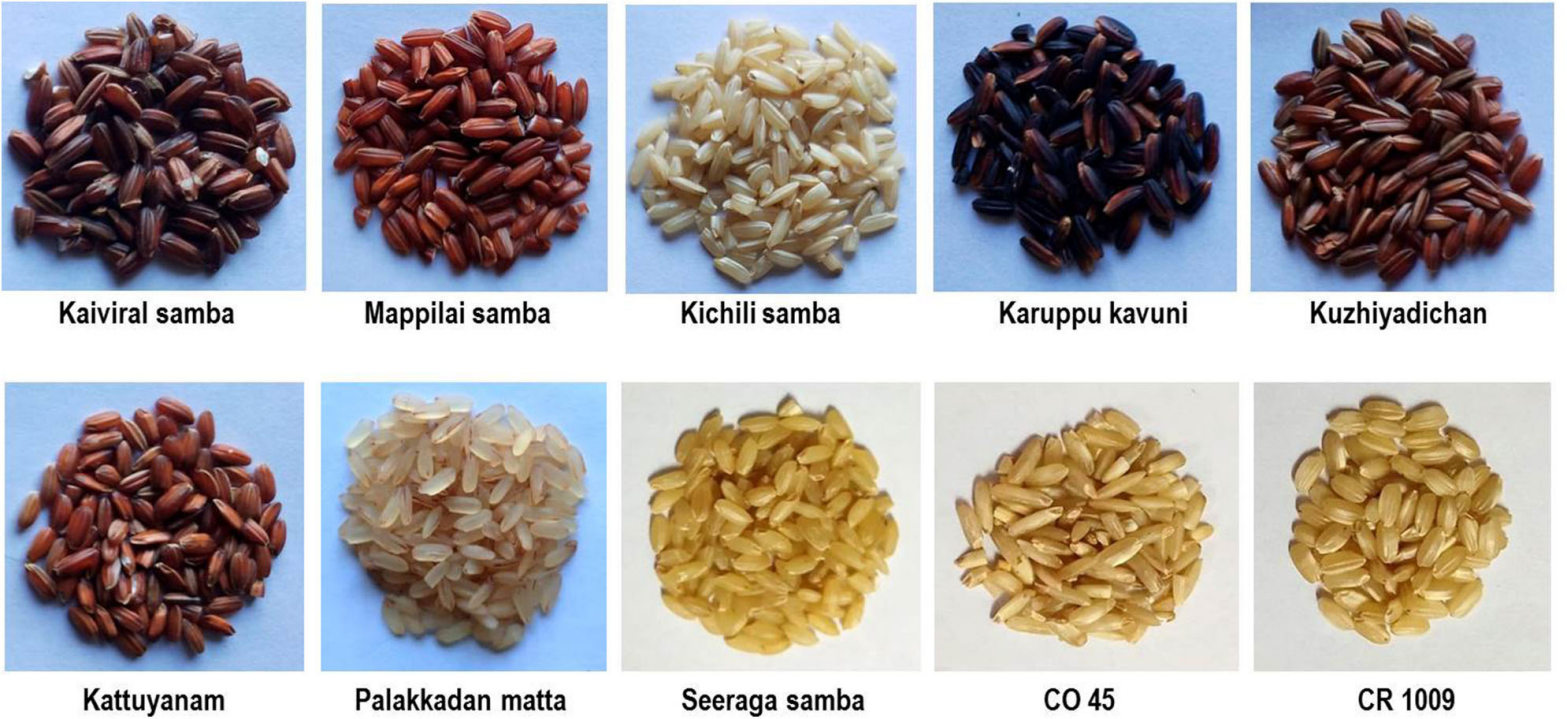
Phenotypic difference of grain color of traditional and modern rice varieties.

The grains of eight traditional rice varieties, namely, Kichili samba, Seeraga samba, Kaiviral samba, Mappilai samba, Karuppu kavuni, Kattuyanam, and Kuzhiyadichan, were procured from CREATE-Nel Jeyaraman Organic Farm & Traditional Paddy Research Center, Kudavasal, Thiruvarur district of Tamil Nadu and Palakkadan matta was procured from a farmer in the Palakkad district of Kerala, India. Two modern rice varieties, CO 45 and CR 1009, were obtained from certified seed-producing farmers of the Thanjavur district, Tamil Nadu, India. These eight traditional and two modern rice varieties were chosen based on the consumers' preference and popularly cultivated in southern India. All the rice varieties were cultivated under a lowland production system following all the agronomic practices adopted for rice cultivation in southern India. The harvest of two biological replicates was performed after reaching proper physiological maturity of grains. In each replication, 500 g of grains were cleaned and subjected to sun drying until the moisture content reached 12.0%. Three technical replicates were performed in each biological replicate for further analysis. The phenotypic difference of grains of traditional and modern rice varieties is shown in Figure 1.

### Total Phenolic Content

The amount of TPC in the extracts was determined by the Folin–Ciocalteu colorimetric method (22). Around 50 g of dehulled rice grains of each variety were ground individually into a fine powder (~20 μm) by a blender; 500 mg of the powdered samples were homogenized with 5 ml of 80% ethanol. The homogenate was centrifuged at 5,000 rpm for 20 min. The supernatant was saved, and the residue was re-extracted five times to the volume of 80% ethanol and recentrifuged. The supernatant was collected and pooled together before evaporating to dryness. The resulting residue was dissolved in 5 ml of distilled water. An aliquot of 0.1 ml of this sample was pipetted out into a test tube, and the volume was made up to 3 ml with distilled water. The Folin–Ciocalteau reagent (0.5 ml) and 2 ml of 20% sodium carbonate solution were added to each tube. Then, the reaction mixture was thoroughly mixed and kept in boiling water for 1 min. After cooling, the absorbance was measured at 765 nm against a reagent blank using a UV BioPhotometer (Eppendorf, Germany). A linear standard curve was constructed by gallic acid (1 mg/1 ml) with the range of 50–300 μg, and the regression equation was *y* = 0.0041*x* – 0.0113 with coefficient of regression (*R*^2^) value 0.9997. Triplicate analysis was performed for each biological replicate of the samples. TPC was expressed as gallic acid equivalent per gram of dry weight of grains.

### Sample Preparation for Volatile Organic Compound Analysis

One gram of finely powdered sample was taken in a 20-ml centrifuge tube, and 10 ml of HPLC-grade ethanol was added into the tube and mixed at 2,000 rpm for 10 min using a vortex mixer (Remi Electrotehnic Limited, Maharashtra, India). The mixture was centrifuged at 5,000 rpm for 20 min (16). The supernatant was concentrated by a rotary evaporator and filtered using a 0.2-μm PVDF syringe filter. The filtrate was stored in a sealed glass vial at 4°C for further GC-MS analysis.

### Chromatography Condition and Analysis

The quantitative analysis of volatile compounds was carried out through GC coupled with a Shimadzu MS (GC-MS-QP2020 NX SHIMADZU). The GC was equipped with a fused silica capillary column DB-5 ms (30 m, 0.25 mm ID) with a film thickness of 0.25 μm. Helium was used as carrier gas at a flow rate of 1.0 ml/min. Then, 1 ml of the sample was kept in a 2-ml screw-top vial in an autoinjector, and 1 μl of the sample was injected in split mode (1:10) (23). The detector and injector temperature was maintained at 250°C. The oven temperature was programmed at 70°C for 15 min, then 30°C/min to 280°C (10 min hold). The MS conditions were electron energy 70 eV, electron impact (EI) ion source temperature 260°C, and transmission line temperature 280°C The mass scan range (*m*/*z*) was 50–650 amu, and data was acquired at full scan mode with solvent delay for 3 min. Total GC run time consisted of 39 min (23). The qualitative analysis of the ethanolic extract was carried using a Shimadzu GC-MS solution™ Ver.4 software.

### Identification of Volatile Constituents

The compounds were identified by the presence of selected ions and by comparing retention index under similar programmed oven temperature conditions for the homologous series of *n*-alkanes (C8–C20) (Sigma-Alrich, Mumbai, India). Identification of individual volatile constituents was based on comparison of mass spectra with those present in the National Institute of Standards and Technology (NIST) and Wiley libraries. The individual volatile compound concentration expressed as percent peak was relative to the total peak area from GC-MS analysis of the sample.

### Statistical Analysis

The results reported in this study are the mean values of at least three technical replications for two biological replications (*n* = 6) unless otherwise stated. Technical repeats were averaged for each biological replicate. Statistical analysis was conducted to study the significant effects of various parameters of selected rice varieties using analysis of variance (ANOVA) and Duncan's multiple range test (DMRT) at the 0.05 level of significance. The agglomerative hierarchical clustering (AHC) was used to understand the relationship between the populations based on volatile organic compounds (VOCs). Euclidean distance was selected to measure the dissimilarity, and Ward's method was used for cluster description. Biplot principal component analysis (PCA) and AHC were performed using the Excel program plug-in XLSTAT version 2020.3.1.

## Results

### Variability in Yield and Total Phenolic Content

In this study, eight traditional and two modern rice varieties of southern India were chosen for assessing the variability of yield, TPC, and grain phytochemical constituents. The present study revealed that the pigmented traditional varieties recorded higher plant height and 1,000 grain weight (g). The modern rice varieties (CO 45 and CR 1009) had recorded 2-fold higher yield than the traditional varieties (Table 1). However, among the traditional varieties, Seeraga samba had documented the highest yield potential (3,705 kg/ha). The total phenolic content ranged from 0.00 to 166.67 μg/g. Interestingly, the total phenolic content observed in the pigmented traditional rice varieties was absent in non-pigmented traditional and modern varieties (Table 1).

**Table 1:** Biometric traits and total phenolic content of traditional and modern rice varieties.

**Varieties**	**Type**	Duration (days)^*^	Height (cm)^*^	**1,000 grain weight (g)**	Yield (kg/ha)^*^	**Total phenolic content (μg GAE/g)**
**TRADITIONAL VARIETIES**						
Kichili samba	Non-pigment	140 ± 2.0^c^	96 ± 4.0^f^	15.0 ± 1.0^h^	2,779 ± 19.1^d^	0.00 ± 0.0^g^
Seeraga samba	Non-pigment	140 ± 2.0^c^	127 ± 2.5^d^	10.9 ± 0.3^i^	3,705 ± 22.1^c^	0.00 ± 0.0^g^
Kaiviral samba	Pigment	130 ± 1.5^d^	149 ± 3.0^b^	30.7 ± 1.2^a^	1,615 ± 17.8^h^	62.7 ± 0.7^d^
Mappilai samba	Pigment	160 ± 1.0^a^	138 ± 5.0^cd^	31.5 ± 1.3^a^	1,729 ± 15.2^h^	47.0 ± 0.7^e^
Karuppu kavuni	Pigment	120 ± 1.5^e^	142 ± 4.0^bc^	24.0 ± 0.4^d^	1,976 ± 18.4^g^	90.0 ± 1.0^c^
Kattuyanam	Pigment	150 ± 1.0^b^	170 ± 5.5^a^	28.5 ± 0.8^b^	2,223 ± 18.5^f^	166.7 ± 4.5^a^
Kuzhiyadichan	Pigment	90 ± 1.5^f^	88 ± 3.0^g^	26.1 ± 0.5^c^	2,408 ± 10.4^e^	120.0 ± 3.5^b^
Palakkadan matta	Pigment	120 ± 1.0^e^	100 ± 3.0^ef^	21.5 ± 0.5^f^	1,852 ± 21.9^h^	16.7 ± 0.5^f^
**MODERN VARIETIES**						
CO 45	Non-pigment	130 ± 2.0^d^	80 ± 3.0^gh^	15.8 ± 0.3^g^	5,800 ± 17.7^a^	0.0 ± 0.0^g^
CR 1009	Non-pigment	160 ± 1.5^a^	106 ± 4.0^e^	22.1 ± 0.4^e^	5,300 ± 13.4^b^	0.0 ± 0.0^g^
CV	–	5.48	3.48	1.80	1.70	2.85
CD (0.05)	–	3.86	8.94	0.94	16.89	3.05

* *Data obtained from farmers in southern India and http://www.agritech.tnau.ac.in/expert_system/paddy/TNvarieties.html. Values are mean ± standard deviation. Data were statistically analyzed using ANOVA. Within columns, means followed by the same letter (a–i) are not significantly different according to Duncan's multiple range test (p < 0.05)*.

### Variability in Volatile Constituents

The variability of phytochemical constituents of the ethanolic extract of all the 10 rice varieties were investigated by the GC-MS analyzer. The chemical profile of VOCs from the eight traditional and two modern varieties showed the presence of 31 constituents being listed out, and its odor typicity was summarized from the literature (Table 2). The identified volatile constituents were summarized into fatty acids (seven), alkanes (seven), phenols (five), alkene hydrocarbons (three), aliphatic alcohols (two), esters (two), terpenes (two), amide (one), benzene (one), and phytosterol (one). The total VOCs comprised about 83.4–97.6% of the total compounds identified (Table 2).

**Table 2:** List of volatile compounds identified in 10 rice southern India popular traditional and modern rice varieties.

**Sr. no**.	**Compound name**	**RT^a^**	**RI^b^**	**Odor description**	**References**
1. Alkane					
1	Dodecane	7.02	1,285	Gasoline-like	(24)
2	Tetradecane	10.35	1,443	Gasoline-like	(24)
3	Hexadecane	16.03	1,612	–	(25, 26)
4	Octadecane	20.77	1,810	–	(25)
5	Heptadecane	22.86	1,852	–	(24)
6	Non-adecane	23.20	1,910	Sweet, rosy	(24)
7	Heneicosane	27.70	2,109	–	
2. Alkene					
1	Tetradecene	10.31	1,413	Mildly pleasant	(24, 26)
2	Pendadecene	12.15	1,512	–	–
3	1-Heptadecene	19.11	1,701	–	(25)
3. Terpene					
1	Azulene	9.91	1,299	–	(24)
2	Squalene	41.70	2,914	Fish oil-like odor	(16, 27)
4. Alcohol					
1	2-Isopropyl-5-methyl-1-heptanol	4.02	1,165	Green	(28)
2	Longiborneol	15.41	1,581	–	–
5. Amide					
1	Arachidonic amide	25.34	2,104	–	–
6. Benzene					
1	Benzonitrile	16.28	1,619	–	(29)
7. Ester					
1	Methyl 9-tetradecenoate	17.89	1,688	Fatty waxy petal	(30)
2	Methyl 4,4-difluororetinoate	31.81	2,144	–	–
8. Phenol					
1	3,5-Di-*tert*-butylphenol	13.68	1,555	–	–
2	2,4-Di-*tert*-butylphenol	14.03	1,555	–	(31)
3	*p*-Cresol	17.88	1,668	–	–
4	Ionol	20.96	1,843	–	(32)
5	2,6-Di-*tert*-butylphenol	41.80	3,315	–	–
9. Fatty acid					
1	Palmitic acid	23.06	1,878	–	(33)
2	Methyl stearate	23.82	2,077	–	–
3	Linoleic acid	24.47	2,093	–	(34)
4	Elaidic acid	32.41	2,175	–	–
5	Stearic acid	34.73	2,177	–	(33, 34)
6	Oleic acid	38.81	2,185	–	(34)
7	Butyric acid	40.30	2,313	Cheesy	(35)
10. Phytosteroid					
1	Stigmast-5-en-3-ol	41.91	4,469	–	(36)

–, not reported;

a RT, retention time;

b *RI, retention index*.

Among the VOCs, the total fatty acids dominated with 46.9–76.3% followed by phenols (2.2–36.1%), terpenes (18.0–32.2%), alkanes (0.5–10.7%), aliphatic alcohols (0.8–5.9%), and alkenes (1.0–4.9%) (Table 3). Palmitic acid (11.1–33.7%), elaidic acid (6.1–31.1%), and linoleic acid (6–28%) were identified as the major fatty acid constituents present in all the 10 varieties of rice. The other fatty acids present in substantial amounts were oleic acid (1.4–17.9%), butyric acid (1.3–3.5%), and stearic acid (1.1–1.9%). The highest linoleic acid (28.0%) was noted in the pigmented traditional variety, Kaiviral samba, which was about 2-folds higher than that found in non-pigmented traditional as well as modern rice varieties. The same variety also recorded ~3-folds of oleic acid concentration, the highest (17.9%) that was found than in all the other varieties. Higher palmitic acid was observed in the non-pigmented modern rice varieties (28.7–33.7%) than that found in both pigmented and non-pigmented traditional varieties (11.1–17.3%). Among the five phenolic components, *p*-cresol is predominant and is only present in the pigmented traditional rice varieties. However, a major terpene component, azulene (12.6–30.7%), was accumulated only in the non-pigmented varieties and was absent in pigmented traditional varieties. Another triterpene compound, squalene, was also present only in the non-pigmented rice varieties in substantial concentrations (Table 3).

**Table 3:** Chemical composition of selected southern India popular traditional and modern rice varieties.

**No**.	**Compound name**	**Area %**											
		**Traditional aromatic varieties**									**Modern varieties**		
		**Kaiviral samba**	**Kichili samba**	**Mappilai samba**	**Karuppu kavuni**	**Kattuyanam**	**Kuzhiyadichan**	**Palakkadan matta**	**Seeraga samba**	**Average**	**CO 45**	**CR 1009**	**Average**
C1.	2-Isopropyl-5-methyl-1-heptanol	–	1.6 ± 0.1^c^	–	–	–	–	–	2.2 ± 0.0^a^	1.9 ± 0.1	1.0 ± 0.0^d^	1.8 ± 0.0^b^	1.4 ± 0.1
C2.	Dodecane	0.6 ± 0.0^a^	–	–	–	0.5 ± 0.0^a^	–	0.5 ± 0.0^a^	–	0.5 ± 0.1	–	–	–
C3.	Azulene	–	12.6 ± 0.3^d^	–	–	–	–	–	30.7 ± 2.5^a^	14.4 ± 0.3	16.9 ± 1.5^c^	29.7 ± 2.7^b^	23.3 ± 0.5
C4.	Tetradecene	–	1.8 ± 0.1^b^	–	–	–	–	–	–	0.9 ± 0.1	1.0 ± 0.0^c^	2.1 ± 0.1^a^	1.6 ± 0.1
C5.	Tetradecane	–	–	1.1 ± 0.0^c^	–	–	–	0.3 ± 0.0^d^	1.8 ± 0.0^b^	0.8 ± 0.1	2.7 ± 0.1^a^	–	2.7 ± 0.2
C6.	Pendadecene	–	1.1 ± 0.1^c^	1.7 ± 0.1^b^	–	–	–	–	1.0 ± 0.0^d^	1.0 ± 0.1	–	1.9 ± 0.0^a^	1.9 ± 0.1
C7.	3,5-Di-*tert*-butylphenol	0.5 ± 0.0^a^	–	–	–	–	–	–	–	0.0 ± 0.0	–	–	–
C8.	2,4-Di-*tert*-butylphenol	2.7 ± 0.1^b^		2.3 ± 0.1^c^	–	2.6 ± 0.1^b^	3.2 ± 0.0^a^	–	–	2.7 ± 0.1	–	–	–
C9.	Longiborneol	0.8 ± 0.1^b^	–	–	–	–	–	–	3.7 ± 0.1^a^	2.3 ± 0.3	–	–	–
C10.	Hexadecane	0.5 ± 0.0^d^	6.1 ± 0.2^b^	2.2 ± 0.1^c^	7.6 ± 0.2^a^	–	–	–	2.1 ± 0.0^c^	3.7 ± 0.3	2.0 ± 0.1^c^	–	2.0 ± 0.1
C11.	Benzonitrile	–	–	36.4 ± 1.5^a^	–	–	–	–	–	18.2 ± 0.2	–	–	-
C12.	*p*-Cresol	13.7 ± 0.4^d^	–	–	24.4 ± 0.9^b^	24.7 ± 0.8^b^	26.1 ± 1.5^a^	22.4 ± 1.5^c^	–	22.3 ± 0.5	–	–	–
C13.	Methyl 9-tetradecenoate	–	–	–	–	–	2.84 ± 0.0	–	–	1.4 ± 0.1	–	–	–
C14.	1-Heptadecene	–	0.9 ± 0.1^b^	1.0 ± 0.0^a^	–	–	–	–	–	0.6 ± 0.3	0.9 ± 0.0^b^	0.9 ± 0.0^b^	0.9 ± 0.1
C15.	Octadecane	–	2.8 ± 0.2^a^	1.3 ± 0.1^c^	1.0 ± 0.0^d^	–	–	–	1.3 ± 0.0^c^	1.3 ± 0.1	2.1 ± 0.1^b^	0.8 ± 0.0^e^	1.5 ± 0.1
C16.	Ionol	0.5 ± 0.0^d^	–	–	0.9 ± 0.0 ^b^	1.0 ± 0.0 ^a^	1.0 ± 0.0 ^a^	0.8 ± 0.0 ^c^	–	0.8 ± 0.1	–	–	
C17.	Heptadecane	0.5 ± 0.0^b^	–	–	–	–	–	0.6 ± 0.0^a^	–	0.6 ± 0.1	–	–	
C18.	Palmitic acid	13.5 ± 0.3^e^	17.3 ± 0.4^c^	11.9 ± 0.6^h^	12.5 ± 0.4^g^	13.0 ± 0.4^f^	11.1 ± 0.3^i^	13.1 ± 0.8^f^	15.6 ± 0.6^d^	13.5 ± 0.3	28.7 ± 1.5^b^	33.7 ± 2.5^a^	31.2 ± 0.5
C19.	Non-adecane	–	–	–	–	–	–	–	–	–	–	1.5 ± 0.1	1.5 ± 0.1
C20.	Methyl stearate	0.6 ± 0.1^i^	1.1 ± 0.1^e^	–	1.2 ± 0.0^d^	1.8 ± 0.0^a^	1.0 ± 0.0^f^	1.5 ± 0.0^c^	0.8 ± 0.0^h^	1.1 ± 0.1	0.9 ± 0.0^g^	1.6 ± 0.1^b^	1.3 ± 0.1
C21.	Linoleic acid	28.0 ± 0.5^a^	10.0 ± 0.2^e^	10.0 ± 0.4^e^	17.5 ± 0.8^d^	17.6 ± 0.7^b^	20.0 ± 1.0^b^	19.1 ± 1.0^c^	12.4 ± 0.8^f^	16.8 ± 0.4	8.5 ± 0.3^g^	6.0 ± 0.3^h^	7.3 ± 0.2
C22.	Arachidonic amide	–	–	–	0.4 ± 0.0^a^	0.3 ± 0.0^b^	0.4 ± 0.0^a^	0.3 ± 0.0^b^	–	0.3 ± 0.1	–	–	–
C23.	Heneicosane	–	–	–	2.0 ± 0.0^a^	–	–	1.8 ± 0.0^b^	–	1.3 ± 0.1	–	–	–
C24.	Retionoic acid	–	–	–	–	–	–	0.4 ± 0.0	–	0.2 ± 0.0	–	–	–
C25.	Elaidic acid	15.2 ± 0.3^g^	14.5 ± 0.3^f^	18.5 ± 1.1^e^	23.7 ± 1.1^d^	27.5 ± 1.5^b^	24.4 ± 1.5^c^	31.1 ± 2.0^a^	14.8 ± 0.6^f^	21.2 ± 0.6	7.8 ± 0.3^h^	6.1 ± 0.2^i^	7.0 ± 0.2
C26.	Stearic acid	1.1 ± 0.1^b^	–	–	–	–	–	–	–	1.1 ± 0.1	1.9 ± 0.1^a^	–	1.9 ± 0.1
C27.	Oleic acid	17.9 ± 0.3^a^	2.9 ± 0.1^f^	5.2 ± 0.3^c^	1.5 ± 0.0^g^	–	–	1.4 ± 0.0^h^	3.6 ± 0.1^e^	5.4 ± 0.3	6.4 ± 0.2^b^	4.0 ± 0.1^d^	5.2 ± 0.1
C28.	Butanoic acid	–	3.5 ± 0.1^a^	1.3 ± 0.0^b^	–	–	–	–	–	1.6 ± 0.1	–	–	–
C29.	Squalene	–	5.4 ± 0.2^b^	–	–	–	–	–	2.5 ± 0.0^c^	2.6 ± 0.2	6.2 ± 0.2^a^	–	6.2 ± 0.1
C30.	2,6-Di-*tert*-butylphenol	2.9 ± 0.1^d^	–	–	–	6.3 ± 0.0^a^	5.7 ± 0.1^b^	4.6 ± 0.2^c^	–	4.9 ± 0.4	-	–	–
C31.	Stigmast-5-en-3-ol	–	–	–	–	–	–	–	–		4.5 ± 0.2	–	4.5 ± 0.2
	Total fatty acids	76.3 ± 1.7^a^	49.3 ± 1.0^g^	46.9 ± 2.0^i^	56.4 ± 1.0^d^	59.9 ± 1.3^c^	56.5 ± 1.1^d^	66.2 ± 1.4^b^	47.2 ± 1.0^h^	57.3 ± 2.3	54.2 ± 1.1^e^	51.4 ± 1.2^f^	52.8 ± 1.3
	Total terpenes	–	18.0 ± 0.7^d^	–	–	–	–	–	33.2 ± 0.9^a^	25.6 ± 1.3	23.1 ± 0.8^c^	29.7 ± 0.9^b^	26.4 ± 0.3
	Total phenols	20.3 ± 0.7^e^	–	2.2 ± 0.1^f^	25.3 ± 1.0^d^	34.6 ± 1.1^b^	36.1 ± 0.8^a^	27.8 ± 1.3^c^	–	24.4 ± 1.2	–	–	–
	Total alkanes	1.6 ± 0.2^f^	10.7 ± 0.5^a^	3.5 ± 0.2^d^	10.6 ± 0.4^a^	0.5 ± 0.1^g^	–	2.9 ± 0.1^e^	3.4 ± 0.1^d^	4.7 ± 0.3	5.1 ± 0.2^b^	4.4 ± 0.2^c^	4.8 ± 0.3
	Total alkenes	–	3.8 ± 0.2^b^	2.7 ± 0.1^c^	–	–	–	–	1.0 ± 0.1^e^	2.5 ± 0.1	1.9 ± 0.1^d^	4.9 ± 0.2^a^	3.4 ± 0.1
	Total aliphatic alcohols	0.8 ± 0.1^e^	1.6 ± 0.1^c^	–	–	–	–	–	5.9 ± 0.2^a^	2.8 ± 0.1	1.0 ± 0.1^d^	1.8 ± 0.1^b^	1.4 ± 0.1
	Others	–	–	36.4 ± 1.0^a^	0.4 ± 0.0^e^	0.3 ± 0.1^f^	3.2 ± 0.1^c^	0.7 ± 0.1^d^	–	8.2 ± 0.2	4.5 ± 0.2^b^	–	4.5 ± 0.3
	Total compounds (%)	99.0 ± 1.1^a^	83.4 ± 1.0^j^	91.7 ± 1.2^g^	92.7 ± 1.2^e^	95.3 ± 1.0^d^	95.8 ± 1.3^c^	97.6 ± 1.5^b^	90.7 ± 1.2^h^	93.3 ± 1.0	89.8 ± 1.4^i^	92.2 ± 1.1^f^	91.0 ± 1.3

*–, not detected at <0.1% by GC-MS analysis; data is mean ± standard deviation (n = 6); within rows, means followed by the same letter (a–i) are not significantly different according to Duncan's multiple range test (p < 0.05)*.

In the present study, seven alkane and three alkene components were identified, and among them, hexadecane is the major alkane constituent (0.5–7.6%), followed by octadecane, tetradecane, heneicosane, non-adecane, and dodecane. Among the alkene compounds, tetradecene was the predominant followed by pendadecene and 1-heptadecene. Two aliphatic alcohols, longiborneol (0.8–3.7%) and 2-isopropyl-5-methyl-1-heptanol (1.0–2.2%), were documented among the varieties. The non-pigmented traditional variety of Seeraga samba recorded the higher aliphatic alcohol longiborneol (3.7%).

### Principal Component Analysis and Cluster Analysis

**Figure 2:**
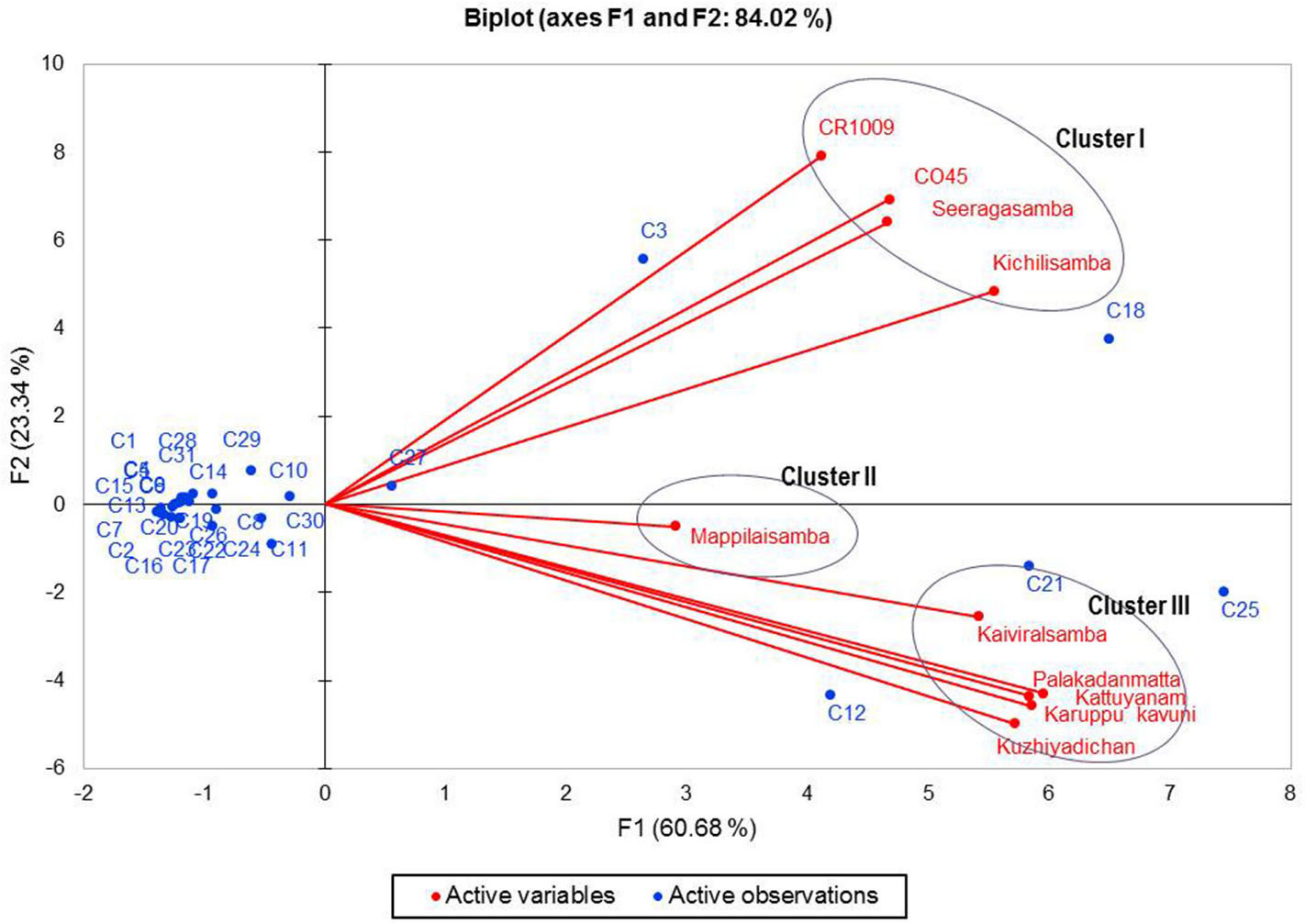
Biplot principal component analysis (PCA) showing correlations of 10 rice samples. Axes (F1 and F2 factors, the first and second principal components, respectively) refer to the ordination scores obtained from the variables (samples) and observations (chemical compounds: C1–C31 from Table 1).

The biplot PCA was performed with the VOCs of the ethanolic extract of the 10 rice varieties. Results showed considerable variability in the chemical composition of VOCs with cumulative variance. PCA constructed the first two principal axes (F1 and F2), displaying 84.02% of the total variance (Figure 2). Also, axes F1 and F2 account for 60.68 and 23.34% of the total variance, respectively. The first and second axes accounted for the positive correlation with the dominant VOCs, azulene, *p*-cresol, palmitic acid, linoleic acid, elaidic acid, and 2,6-di-*tert*-butylphenol. The second axis accounted for the negative correlation of oleic acid, palmitic acid, and azulene.

**Figure 3:**
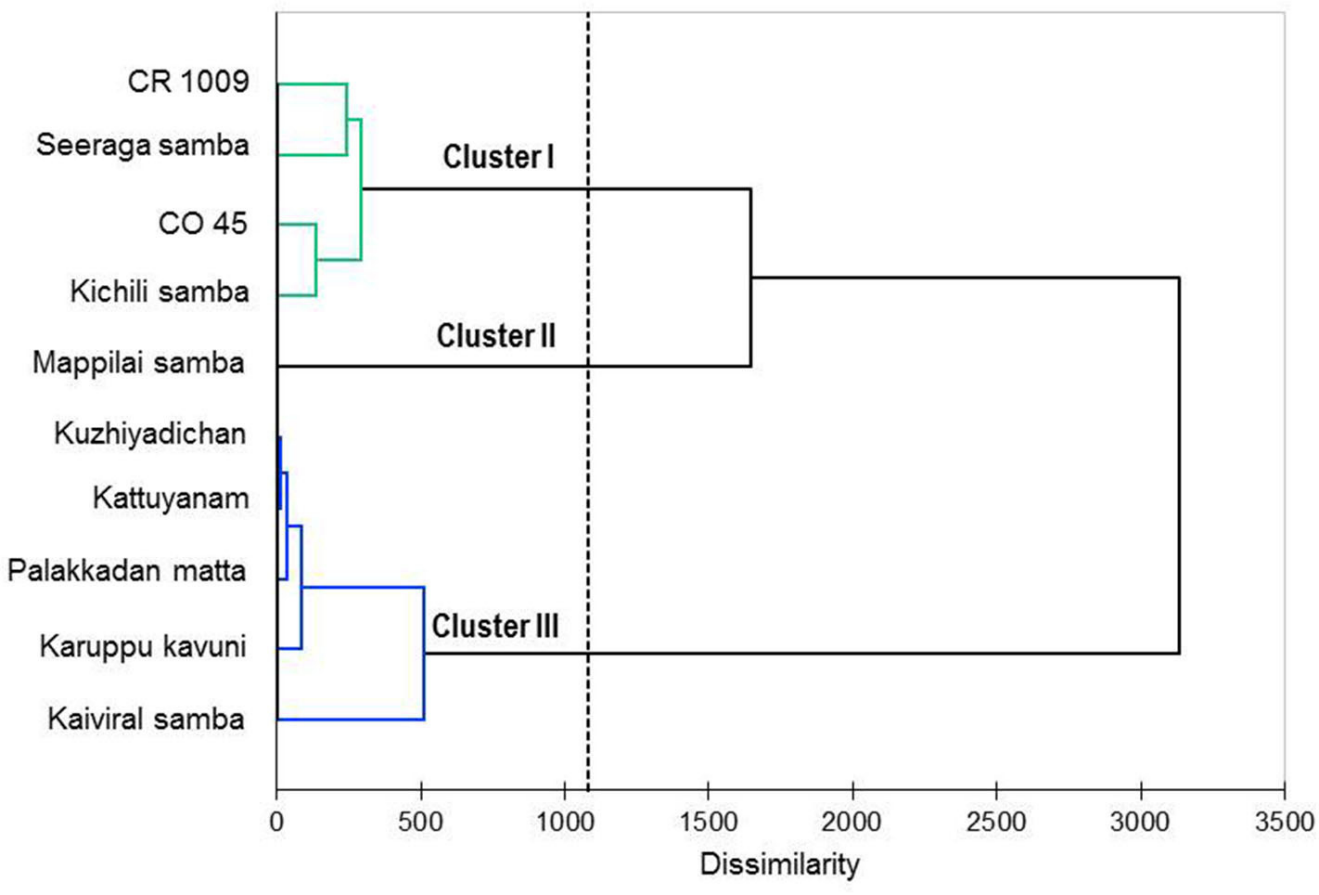
Dendrogram obtained by agglomerative hierarchical cluster analysis of the volatile constituents of the traditional and modern rice varieties under study based on Ward's method using the Euclidean distances.

**Figure 4:**
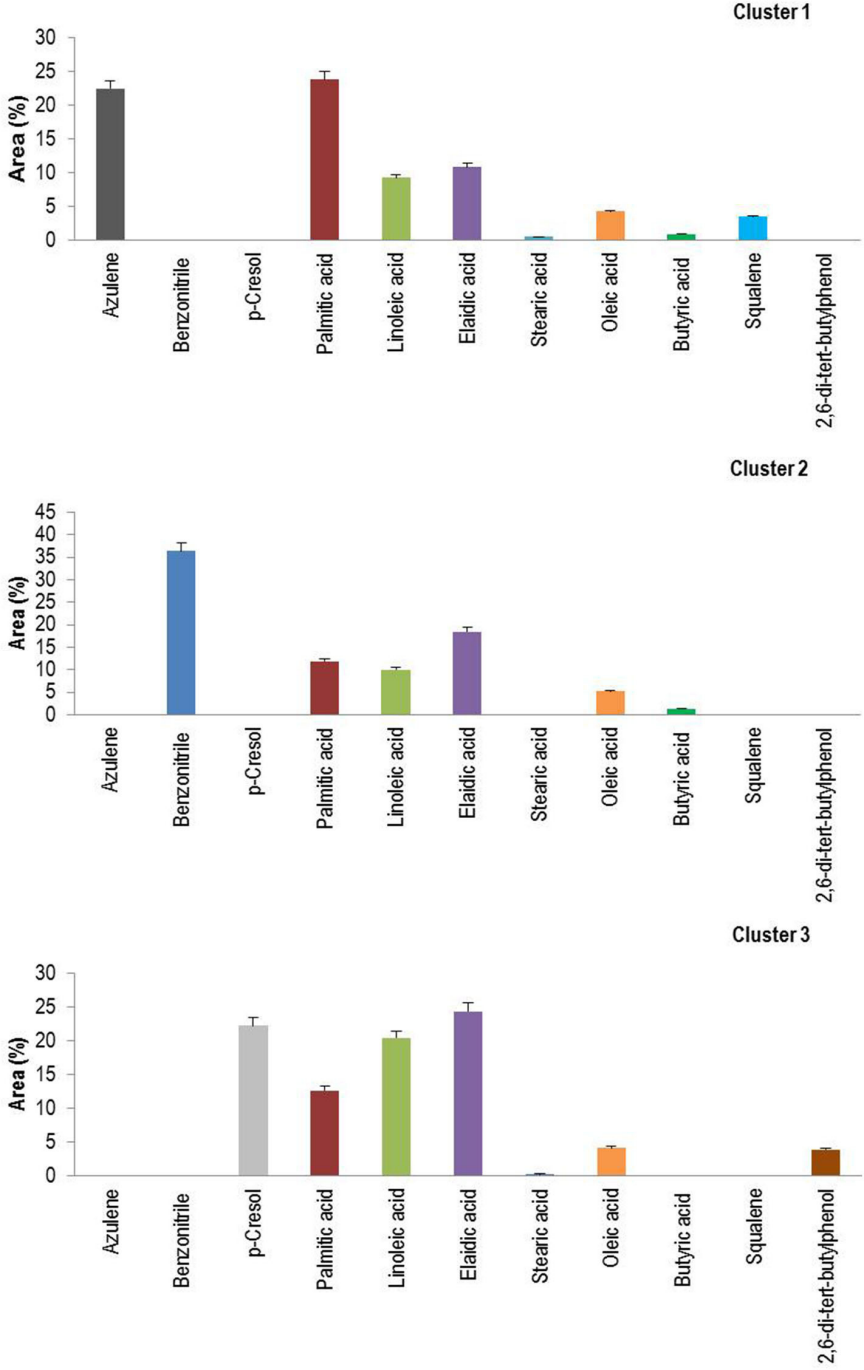
Clusters means of the major volatile organic compounds of rice varieties.

The agglomerative hierarchical cluster analysis was performed on the VOCs of eight traditional (six pigmented and two non-pigmented) and two non-pigmented modern rice varieties, and the dendrogram showed three main clusters (Figure 3). Cluster 1 formed four varieties that are non-pigmented grain types, namely, CR 1009, CO 45, Seeraga samba, and Kichili samba. Cluster 2 is composed of one pigmented variety (Mappilai samba), and cluster 3 is made up of five pigmented traditional varieties (Kaiviral samba, Karuppu kavuni, Kattuyanam, Kuzhiyadichan, and Palakkadan matta). Varieties belonging to the same group show more similarity in the composition of VOCs. In brief, azulene, benzonitrile, *p*-cresol, palmitic acid, linoleic acid, elaidic acid, oleic acid, stearic acid, butyric acid, squalene, and 2,6-di-*tert*-butylphenol were found in higher concentrations in the VOCs and formed three chemical clusters (Figure 4). The varieties under cluster 1 had higher azulene, squalene, palmitic acid, and oleic acid, while the variety Mappilai samba had higher benzonitrile concentration in cluster 2. The varieties under cluster 3 had a higher concentration of *p*-cresol, elaidic acid, and 2,6-di-*tert*-butylphenol (Figure 4).

## Discussion

The nutritional quality of traditional varieties is higher than that of the grain produced by modern rice varieties, owing to their additional accumulation of biologically active chemical compounds and pigmentations in grains (7, 37). Evaluation of biometrical traits like plant height, number of tillers, panicle length, 1,000 grain weight, and other traits is essential for developing a variety with a higher yield. No systematic study is available on the yield-attributing traits in South Indian traditional rice landrace/varieties. These varieties are being considered as low yielders. Yield achievements and rice production in many countries are very significant and record breaking (38). At the same time, the native nutrition and grain quality are ignored since the inception of the Green Revolution. The latter is essential in the current scenario of rice breeding. The present study observed that the traditional varieties of Mappilai samba, Karuppu kavuni, Kaiviral samba, and Kattuyanam had recorded higher plant height (138–170 cm). Hence, farmers can use these varieties for dual purposes like the production of fodder and grain yield. Also, Kuzhiyadichan has a duration of 90 days and is closer to modern varieties in terms of earliness; therefore, farmers can choose this variety for a short-duration cropping pattern.

Genetic variability for biometric and TPC traits indicated that diverse taste preferences are followed in South India. In the present study, the TPC for 1 g of rice extract is expressed as gallic acid equivalents and was largely accumulated in pigmented traditional rice. The highest concentration of TPC was recorded in Kattuyanam among the pigmented rice varieties. According to Krishnanunni et al. (16), the pigmented traditional variety Mappilai samba recorded 47.35 μg gallic acid equivalents (GAE)/g of TPC and was within the range as found in our present study, i.e., 47.01 μg GAE/g. Earlier studies have also identified that the pigmented rice grains accumulated TPC and various phenolic compounds (15). The concentration of TPC is positively associated with antioxidant activity that protects humans from diabetes and cardiovascular diseases (15–18).

Profiling the VOCs of grains of traditional as well as modern varieties is essential not only to be utilized in breeding programs but also to ensure rice grain quality in the new food market. Among the 31 VOCs identified in the eight traditional and two modern rice varieties, the majority of them belonged to fatty acids, alkanes, and phenols. In earlier studies, several components were already identified and some of the compounds' odor typicity was also reported (Table 2). However, to our knowledge, some of the compounds were newly identified in the present study, which are heneicosane, pendadecene, longiborneol, arachidonic amide, 2,6-di-*tert*-butylphenol, 3,5-di-*tert*-butylphenol, *p*-cresol, methyl stearate, and elaidic acid. Further studies need to be conducted for the discovery of the biological activities of these new compounds.

The presence of *p*-cresol, hecaxadecane, arachidonic amide, and elaidic acid might be the reason for the attractiveness of Karuppu kavuni among consumers and it is thus considered as ideal for festive occasions in India. Trace levels of octanal and linalool were not detected in any of the selected southern Indian rice varieties that are tested in this study. However, these VOCs were previously identified in exotic rice varieties (21). Benzonitrile was predominantly accumulated only in Mappilai samba and not in other rice varieties. Furthermore, a phytosterol compound, stigmast-5-en-3-ol, was only present in the modern rice variety, CO 45, which was absent in all the other varieties. In the present study, squalene ranged from 2.5 to 6.5%, which was higher than the value of 1.5–3.5% found in an earlier report (16). The major triterpene compound, azulene, was predominantly accumulated in the non-pigmented rice variety Seeraga samba, followed by CR 1009, CO 45, and Kichili samba. The results of the present study are in agreement with the earlier reports for the similar accumulation of alkanes, alkenes, amides, and aliphatic alcohols in the rice grains (16). Moreover, the potential known health benefits of major VOCs are well-described in Table 4.

**Table 4:** Major volatile organic compounds (VOCs) of southern India popular traditional and modern rice varieties and their potential health benefits.

**Sl. no**.	**Major VOCs**	**Target/model**	**Known health effects**	**References**
1	Azulene	Clinical trials with four females and two males	Anti-inflammatory activity	(39)
2	*p*-Cresol	Butylated hydroxytoluene (BHT)	Antioxidant	(40)
		HT29 and Caco-2 cells	Anticancer activity	(41)
3	Palmitic acid	Not reported	Increases the risk of cardiovascular diseases	(42)
4	Linoleic acid	2–3 g/day	Reduces coronary heart diseases (CHD)	(43)
		Caco-2, BT-474, A-549	Anticancer	(44, 45)
5	Elaidic acid	CP-4126 cell lines	Anticancer	(46)
6	Oleic acid	C6 glioma cells	Reduces cardiovascular risk by reducing blood cholesterol	(47, 48)
7	Squalene	DPPH assay	Antioxidant	(49)
		50 female CD-l mice	Protects against skin tumors	(50)
		>13.5 g/day	Reduces wrinkles and decreases UV-induced DNA damage in human skin	(51)
8	Butyric acid	Mm-A cells	Anticancer	(52)
		HCT-116 and HT-29	Anticancer	(53)
		T (Treg) cells	Gastrointestinal activity	(54)
9	Benzonitrile	DPPH assay	Antioxidant	(55)
10	Stearic acid	MDA-MB-361, MCF-7, and MDA-MB-231	Reduces human breast cancer	(56)
		11 human (males)	Lowers LDL cholesterol concentrations	(57)
11	2,4-Di-*tert*-butyl phenol	HeLa cells (IC_50_: 10 μg/ml)	Cytotoxicity	(58)
		DPPH assay	Antioxidant	(58)
		*Aspergillus niger, Fusarium oxysporum*, and *Penicillium chrysogenum*	Antifungal	(58)

Of the fatty acids identified, palmitic acid, linoleic acid, and elaidic acid are present in all the 10 rice varieties. However, the palmitic acid content is seen to be nearly 2-folds higher in modern varieties than in the traditional varieties. A pigmented traditional variety (Kaiviral samba) had the highest levels of ~2-folds of linoleic and oleic acid than those found in the non-pigmented traditional as well as modern varieties. Earlier studies reported that rice bran oil contains 75% of total unsaturated fats (38.4% oleic acid and 34.4% linoleic acid), and these two fatty acids are mainly responsible for lowering the cholesterol level (59, 60). Also, these two natural fatty acids have been extensively studied and are exhibited to reduce coronary heart disease (CHD) and cardiovascular risk (43, 47, 48) as shown in Table 4. Mozaffarian (43) recommended that evidence-based dietary consumption of α-linoleic acid (2–3 g/day) is associated with the prevention of CHD. Thus, the daily consumption of an α-linoleic acid-rich rice variety (Kaiviral samba) could protect people from CHD.

Fattore and Fanelli (42) specified that the intake of high palmitic acid from food sources could increase the risk of cardiovascular diseases (CVD). The present study noted that palmitic acid was two to three times highly accumulated in the grains of non-pigmented modern rice varieties than in pigmented traditional landraces. Hence, the consumption of pigmented traditional rice grains might protect people from CVD. Squalene is a triterpenoid compound present in significant concentrations in the non-pigmented rice varieties Kichilli samba, Seeraga samba, and CO 45. The primary function of squalene is protecting the human skin surface from lipid peroxidation caused by exposure to UV and other ionizing radiation (49). Squalene was also reported to have high potential for antibacterial, anticancer, and immunostimulant activities (49–51) as summarized in Table 4. Among the different alkanes, hexadecane is the predominant one, and it has been extensively studied and has been shown to exhibit antifungal activity against the endophytic fungus Berkleasmium sp. Dzf12 (61).

The grains of Seeraga samba and Kichilli samba traditional rice varieties are also used for the preparation of special foods like biryani (cooked rice with fragrances with preferred vegetables or meats) in South Indian states. The pigmented traditional landrace Mappilai samba rice is popularly cultivated in Tamil Nadu and is consumed for imparting strength (62). Besides, several traditional varieties have been used in South India for preparing various food items like pongal, sarkarai pongal, puttu, idli, dosai, appam, adai, idiyappam, kozhukattai, payasam, semiya uppuma, adirasam, and others. The pigmented traditional rice varieties are also used in preparing food products like cookies, murukku (one of the famous South Indian snacks), and puffed and flaked rice (63). The traditional variety Neelam samba has been used for a long time to balance hormonal levels in lactating mothers (62).

The biplot PCA and AHC analysis were carried out to understand the relationship among the varieties based on the volatile compounds using Euclidean distance assessing divergence between the samples (64). Clusters formed by both PCA and Ward's cluster analysis are the same and it shows three clusters among the rice varieties. Cluster 1 consisted only of non-pigmented rice including modern rice varieties that are rich in palmitic acid, oleic acid, azulene, and squalene. Cluster 2 consisted only of one pigmented rice variety—Mappilai samba—and it accumulated benzonitrile concentration which is absent in other varieties. Cluster 3 contained pigmented rice varieties only and had a higher concentration of *p*-cresol, linoleic acid, elaidic acid, and 2,6-di-*tert*-butylphenol. The existence of *p*-cresol in the traditional pigmented rice variety of Kattuyanam of cluster 3 suggested anticancer activity (41). Several previous studies have already suggested that VOCs of rice have antioxidant, anti-inflammatory, anticancer, and antifungal activities due to the presence of dominant VOCs like linoleic acid, elaidic acid, and 2,6-di-*tert*-butylphenol (43, 44, 46, 58). More interestingly, the cluster based on PCA and Ward statistics showed consistently similar grouping patterns (clusters I, II, and III) with the same varieties, implying the genotypic similarity in terms of VOCs and confirming the association among the traditional and modern varieties. This necessitates further molecular studies to understand the proportion of domesticated genes that causes the genetic similitude among the traditional and modern varieties. At the same time, such genomic studies provide insights of VOC genes and their QTL regions to map traits and the linked markers. The present study opened the knowledge of the variability of VOCs that can pave the way to promote studies that investigate the pharmacological potentials of rice. Furthermore, these findings could be useful for a researcher in the choice of suitable traditional varieties for plant breeding programs like developing varieties with therapeutically and nutritionally valuable VOCs as well as high yield in rice.

## Conclusions

Enhancing the rice crop with grain quality parameters is highly crucial along with improving yield and pest and disease resistance. In order to keep the native fragrances and nutritional values intact, traditional varieties needed to be elaborately studied for their non-targeted traits (other than yield) to explore within-species variability and introgression. The present study is the first study that provided extensive data on the variation of TPC and the composition of VOCs compared to yield among the traditional and modern rice varieties of South India. This characterization of VOCs' composition from different rice varieties (pigmented and non-pigmented) could be useful for further studies, tackling the pharmacological and pesticidal activities of the compounds. We have identified the ethanolic extracts of 10 rice varieties which predominantly consisted of fatty acids followed by alkanes, phenols, alkene hydrocarbons, aliphatic alcohols, esters, terpenes, amides, and others. The results also earmarked that the pigmented traditional rice varieties had the highest phenols, *p*-cresol, linoleic acid, and elaidic acid, whereas white or non-pigmented rice had the highest palmitic acid and azulene. Additionally, the traditional rice variety Kaiviral samba will be a source of linoleic acid and oleic acid, whereas Palakkadan matta is a good source of elaidic acid. GC-MS analysis identified some novel molecules like heneicosane, longiborneol, arachidonic amide, 6-di-*tert*-butylphenol, 3,5-di-*tert*-butylphenol, and *p*-cresol that have been identified in any traditional and modern rice varieties for the first time. These outcomes implied that significant potential traits were retained during the domestication, cultivation, and selective breeding programs. Future investigation could focus on more numbers of popular traditional rice varieties to identify the novel bioactive VOCs, which are mandatory for the new rice industrial arena. The clear understanding of medicinally important VOCs against various diseases by systematic clinical studies will expand the medical benefits and applications of rice VOCs. Additionally, research on the yield and yield-attributing traits and the genotype × environment effect of several South Indian traditional rice varieties has not yet been conducted; hence, future studies also need to investigate the yield and yield-attributing traits of South Indian traditional varieties at different environments for the improvement of rice quality traits. In parallel, modern varieties can be screened for the selected VOCs to fetch the market and consumer preferences.

## Data Availability Statement

The original contributions presented in the study are included in the article/supplementary material, further inquiries can be directed to the corresponding author/s.

## Author Contributions

KA and MG conceptualized the manuscript and wrote the manuscript. KA, SV, and MA performed the experiment. JS and AK collected the samples and performed the data analyses. MG and VS edited and updated the manuscript. All authors contributed to the article and approved the submitted version.

## Conflict of Interest

The authors declare that the research was conducted in the absence of any commercial or financial relationships that could be construed as a potential conflict of interest.
